# Comment on Piña et al. Ten Approaches That Improve Immunostaining: A Review of the Latest Advances for the Optimization of Immunofluorescence. *Int. J. Mol. Sci.* 2022, *23*, 1426

**DOI:** 10.3390/ijms23084372

**Published:** 2022-04-15

**Authors:** Eugene V. Sheval

**Affiliations:** 1 Belozersky Institute of Physico-Chemical Biology, Lomonosov Moscow State University, 119991 Moscow, Russia; sheval_e@belozersky.msu.ru; 2 Department of Cell Biology and Histology, Faculty of Biology, Lomonosov Moscow State University, 119991 Moscow, Russia

With great interest, I have read the article “Ten Approaches That Improve Immunostaining: A Review of the Latest Advances for the Optimization of Immunofluorescence” written by Piña et al. and published in the *International Journal of Molecular Sciences* on 26 January 2022 [1]. The authors described several new methods that overcome the different limitations of immunofluorescent staining and, in particular, discuss the problem of protein detection inside nucleoli using immunofluorescence. The nucleolus is the densest nuclear substructure, and it is formed by three compartments: fibrillar centers, a dense fibrillar component and a granular component. The granular component occupies most of the nucleolar volume, and when detecting granular component proteins using antibodies, in most cases, the entire volume of the nucleolus is stained except for small vacuoles, which correspond to the fibrillar centers. However, some antibodies to the proteins of granular components only label proteins at the nucleolar periphery (rim localization). Previously, we presented data that indicated that this staining pattern may be an artifact related to the low availability of structures with high concentrations of an antigen recognized by antibodies used in immunofluorescence studies [2,3]. It is important to emphasize that our experiments have shown that the obstacle to antibody penetration is not the density of the structure but rather the antigen concentration.

To achieve correct immunostaining, we tried to reduce the antigen concentration using a limited proteolytic treatment of cells fixed with paraformaldehyde. As a result, we developed a protocol that allowed us to detect one of the major nucleolar proteins, B23 (nucleophosmin, NPM1) in the entire volume of the granular component [3]. Modifications of this method have been used in several works with cultured cells [4,5], and our protocol was also adapted to work with murine oocytes [6].

The article by Piña et al. analyzes this approach and describes the protocol in detail [1]. However, when describing the protocol, it states that methanol should be used for fixation. In our work, we indeed describe the case of methanol fixation. However, after methanol fixation, the protein was detected in the inner regions of the nucleus without protease treatment, probably due to partial extraction of the protein. Protease treatment was used for fixation with paraformaldehyde (3.7% paraformaldehyde at room temperature for 10 min), which is important for preserving cell structures (fixation with paraformaldehyde is much better than fixation with methanol in regard to the preservation of cell structures).

In conclusion, it is important to note that published literature has made some important points regarding the artifactuality of peripheral staining of the nucleolus by antibodies. In particular, Stenström et al. presented data on the possible existence of a group of proteins that are indeed localized in the nucleolar rim [7]. Further studies are required to definitively clarify the situation of nucleolar peripheral staining, and it is important to consider different reasons for the low availability of cellular structures to antibodies.
